# Mammary Gland Involution Provides a Unique Model to Study the TGF-β Cancer Paradox

**DOI:** 10.3390/jcm6010010

**Published:** 2017-01-13

**Authors:** Qiuchen Guo, Courtney Betts, Nathan Pennock, Elizabeth Mitchell, Pepper Schedin

**Affiliations:** 1 Department of Cell, Developmental and Cancer Biology, Oregon Health and Science University, Portland, OR 97239, USA; guo@ohsu.edu (Q.G.); bettsc@ohsu.edu (C.B.); pennock@ohsu.edu (N.P.); mitceliz@ohsu.edu (E.M.); 2 Young Women’s Breast Cancer Translational Program, University of Colorado Anschutz Medical Campus, Aurora, CO 80045, USA; 3 Knight Cancer Institute, Oregon Health and Science University, Portland, OR 97239, USA

**Keywords:** weaning-induced mammary gland involution, TGF-β, cellular crosstalk, cancer

## Abstract

Transforming Growth Factor-β (TGF-β) signaling in cancer has been termed the “TGF-β paradox”, acting as both a tumor suppresser and promoter. The complexity of TGF-β signaling within the tumor is context dependent, and greatly impacted by cellular crosstalk between TGF-β responsive cells in the microenvironment including adjacent epithelial, endothelial, mesenchymal, and hematopoietic cells. Here we utilize normal, weaning-induced mammary gland involution as a tissue microenvironment model to study the complexity of TGF-β function. This article reviews facets of mammary gland involution that are TGF-β regulated, namely mammary epithelial cell death, immune activation, and extracellular matrix remodeling. We outline how distinct cellular responses and crosstalk between cell types during physiologically normal mammary gland involution contribute to simultaneous tumor suppressive and promotional microenvironments. We also highlight alternatives to direct TGF-β blocking anti-cancer therapies with an emphasis on eliciting concerted microenvironmental-mediated tumor suppression.

## 1. Introduction

The function of transforming growth factor β (TGF-β) signaling in cancer remains a paradox, as TGF-β has both tumor suppressive and promotional activities [1,2]. The conflicting data as to whether TGF-β acts dominantly as a tumor suppressor or promotor are largely context dependent, that is, contingent on the transformed state of the tumor cell and the constituents of the tumor microenvironment [2]. Tumor cell intrinsic properties that impact TGF-β signaling include genetic alterations in the pathway, however, the local tumor microenvironment plays a central role in determining if a tumor cell is ultimately suppressed or activated by TGF-β. This is because most cell types respond to TGF-β stimulation [3,4,5], and coordinated crosstalk between various cell types is required to integrate TGF-β signaling across the tissue. Furthermore, based on the types of TGF-β responding cells within the tissue, pro- and anti-tumor signals can be generated simultaneously.

In this review, we explore the dual nature of TGF-β function in cancer by investigating several of the myriad roles of TGF-β within the context of normal tissue biology, using weaning-induced mammary gland involution as a model. Weaning-induced mammary gland involution is TGF-β-dependent, with direct roles described for epithelial cell apoptosis and cell clearance [6,7], immune modulation [4], and extracellular matrix remodeling [8]. We argue that exploring tissue level TGF-β biology using this multicellular integrated approach is critical for understanding how to successfully target TGF-β for cancer therapeutics. 

## 2. The TGF-β Cancer Paradox

The TGF-β paradox has been well described in the context of epithelial cells [3,9]. TGF-β is well known to operate through two key pathways, termed the canonical and non-canonical signaling pathways, which have been studied and reviewed intensively elsewhere [10,11,12]. While the molecular activities downstream of these pathways are distinct, their differential employment does not explain the tumor observed TGF-β paradox. In normal epithelium, key tumor suppressive capacities of TGF-β include inhibiting normal epithelial cell proliferation and inducing apoptosis [3], functions thought to be required for blocking progression of initiated cancer cells [13]. Early studies showed that TGF-β treatment of primary, non-transformed rabbit uterine epithelial cells inhibited cell proliferation and induced apoptosis in a concentration dependent manner [14]. Subsequently, researchers discovered that TGF-β inhibits cell proliferation by blocking the late G1 activation of the cyclin-dependent kinases [15] and induces cell apoptosis by upregulating the Death-Associated Protein kinase, a caspase mediated extrinsic apoptotic kinase, which can also be triggered by FAS (tumor necrosis factor receptor superfamily, member 6) and tumor necrosis factor α (TNFα) receptor ligation [16,17,18]. Cell culture studies have shown that TGF-β treatment induced apoptosis in a variety of primary epithelial cells and established immortalized but non-transformed cells, including hepatocytes [19], colonic epithelial cells [20], and mammary epithelial cells [21]. TGF-β also can inhibit carcinogenesis. For example, a mixed strain of Rag2^−/−^ mice (129S6 × CF-1) develops an inflammation associated hyperplasia specific to cecum and colon [22]. In this mouse model, the combination of inflammation associated hyperplasia with loss of TGF-β is sufficient to promote the development of overt colon cancers [22]. Cumulatively, these data support a key role for TGF-β in tumor suppression. 

In contrast to the described tumor suppressive functions of TGF-β, this cytokine has also been shown to support tumor progression through various tumor cell intrinsic mechanisms. With tumor progression, tumor cells can acquire resistance to TGF-β growth inhibition by downregulation or mutation of the TGF-β receptors (TGF-βR) [23] or TGF-β pathway downstream effectors such as Smad4 [24], a coactivator in the canonical TGF-β pathway [25]. In many epithelial cancer cell lines, the inhibitory effects of TGF-β signaling are not simply bypassed, but rather TGF-β signaling becomes cancer promotional. This occurs via TGF-β induction of epithelial-mesenchymal transition (EMT), as measured by downregulation of the epithelial cell markers E-cadherin, and upregulation of the mesenchymal marker vimentin [26]. Furthermore, TGF-β can enhance stemness properties of tumor cells including colony formation, in vivo tumor growth, and chemotherapy drug resistance [27,28]. Additional early evidence that TGF-β facilitates cancer progression was observed in a rat model, where a mammary adenocarcinoma clone of the MTLn3 cell line was injected through the tail vein of syngeneic F344/NHSd female rats. TGF-β pre-treatment of the tumor cells prior to tail vein injection resulted in a three-fold increase in lung metastases [29]. In total, experimental data support the hypothesis that more advanced cancers are promoted by TGF-β signaling, and led to the development of TGF-β inhibitors for cancer therapy. 

Currently, more than 30 anti-TGF-β drugs have been developed and evaluated in preclinical and clinical trials, results of which are excellently reviewed elsewhere [30,31,32,33]. In sum, anti-TGF-β drugs have had limited and sporadic efficacy in clinical cancer treatment [30,31,32,33]. In a phase III clinical study with 270 patients with non-small cell lung cancer, treatment with the TGF-β antisense oligonucleotides drug Belagenpumatucel-L did not impact patients’ progression-free survival or overall survival [33]. Similarly, TβM1, a monoclonal antibody against TGF-β1, showed no effect in patients with metastatic colon and rectal cancers [34]. Importantly, understanding the impact of TGF-β signaling in the context of tumor cell intrinsic biology has not provided a clear explanation for the lack of robust clinical efficacy in these clinical trials. A relatively unexplored explanation for these results is based upon the fact that most cell types in the body respond to TGF-β stimulation. Thus, the resulting cellular crosstalk dramatically complicates the biology and outcome of TGF-β inhibition in cancer patients. To begin to understand this tissue-level complexity and how it can impact clinical efficacy, we discuss TGF-β signaling in the context of weaning-induced mammary gland involution, where TGF-β impacts distinct cell types and their crosstalk to coordinate key biological processes. Many of these biological processes are shared with those characterized in both promotional and suppressive tumor microenvironments, making involution a unique model to shed light on the TGF-β cancer paradox. 

## 3. Mammary Gland Involution as a Model to Integrate Disparate TGF-β Biology

**Figure 1:**
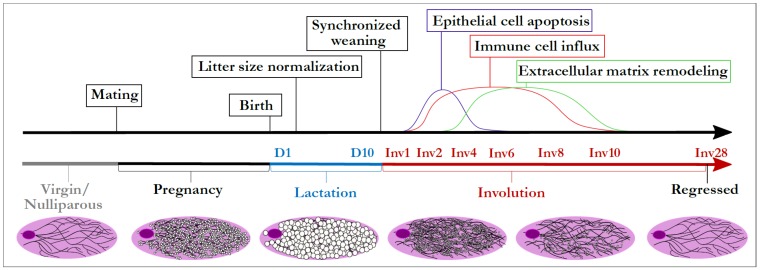
Rodent model of synchronized weaning-induced mammary gland involution. Male and female rodents are mated for 3–5 days. After pup birth, litter size is normalized to ensure equal lactation load per dam, and weaning initiated during peak lactation (9–13 days) by pup removal. The kinetics of three major cellular events during involution are shown in the above timeline: epithelial cell apoptosis, immune cell influx, and extracellular matrix remodeling. Days since start of lactation are indicated by “D” followed by a number, and days since synchronized weaning by “Inv”. Whole gland representations of mammary epithelial ducts (black lines) as well as milk accumulation (white filled circles) are displayed below the corresponding developmental stages in the timeline.

The mammary gland is dynamically regulated by reproductive state. During pregnancy, as the gland prepares for milk production and secretion, the mammary gland expands dramatically through extensive epithelial cell proliferation and differentiation [35]. After lactation, the mammary gland undergoes weaning-induced mammary gland involution, whereby unnecessary secretory epithelial cells are eliminated through a concerted, developmentally regulated cell death and tissue remodeling program [36,37,38,39,40]. Using rodent models, lactational load is normalized and involution synchronized by standardizing pup removal, allowing for the cellular and molecular events of involution to be analyzed in detail (Figure 1). The hallmark features of involution include clearance of ≥80% of the mature, secretory mammary epithelium through programmed cell death [37,41], adipocyte repopulation [42], and immune cell infiltration and extracellular matrix (ECM) deposition consistent with wound healing [43,44,45]. This developmentally regulated window of tissue regression captures the spectrum of TGF-β functions including processes that are conventionally tumor suppressive (epithelial cell apoptosis) as well as tumor promotional (ECM deposition and immune suppression). The cell death that occurs during involution is consistent with tumor suppression, as clearance of early transformed epithelial cells can likely prevent tumor formation [46]. However, the intensive stromal remodeling and immune suppression that occurs with involution is known to promote pre-existing tumor cells [45,47,48]. In fact, the co-existence of both tumor suppressive and promotional attributes within the involuting mammary gland may help explain the dual effect pregnancy has on breast cancer incidence as well as prognosis [46,49], a topic that is addressed more fully below. 

## 4. Evidence for TGF-β Orchestrating Weaning-Induced Mammary Gland Involution

**Figure 2:**
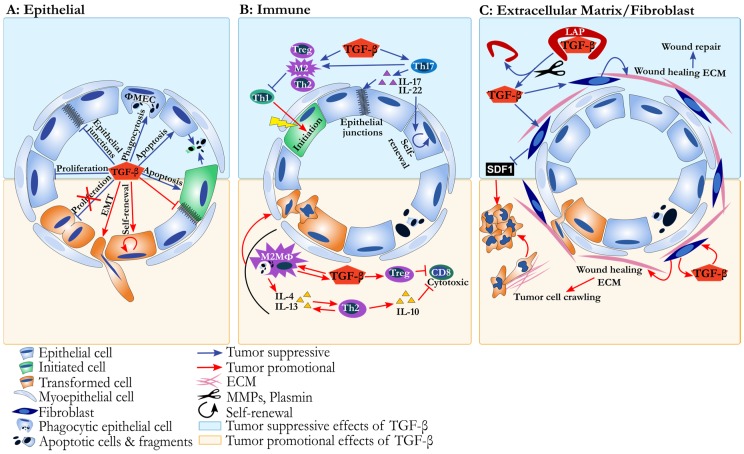
The tumor suppressive and promotional functions of transforming growth factor β (TGF-β) in the involuting mammary microenvironment. Each panel depicts an involuting mammary acini either lacking (top panel) or containing (bottom panel) tumor cells. (**A**) Epithelium: In non-transformed mammary epithelial cells (top half of diagram, blue arrows), TGF-β suppresses cell proliferation, and induces tumor suppressive apoptosis and phagocytosis mediated by loss of epithelial junctions. In the presence of transformed cells (bottom half of diagram, red arrows), TGF-β can promote cancer progression by inducing epithelial mesenchymal transition (EMT) and stem cell phenotypes. Additionally, anti-proliferative functions of TGF-β can be lost in tumor cells via mutations in TGF-β signaling pathways (depicted by red X); (**B**) Immune milieu: In the absence of tumor cells (top half of diagram, blue arrows), TGF-β suppresses chronic inflammation by inducing T-helper 2 (Th2) cells and T-helper 17 (Th17) cells which can suppress T-helper 1 (Th1) cells mediated tumor initiation. This immune environment also maintains epithelial stem cell health and epithelial cell junctional integrity (blue arrows). In the presence of tumor cells (bottom half of diagram, red arrows), TGF-β induced Th2 immunity suppresses anti-cancer CD8 T cell cytotoxic function and directly activates tumor cells through growth factor/cytokine signaling; (**C**) Extracellular matrix/fibroblast: Active TGF-β is released in the extracellular microenvironment when proteases cleave the Latency Associated Peptide (LAP). TGF-β signaling within fibroblasts impairs production of stromal cell-derived factor-1 (SDF1). In the absence of tumor, TGF-β signaling plays a critical role in maintaining tissue integrity (top half of diagram, blue arrows). In the presence of tumor cells (bottom half of diagram, red arrows) a wound healing like extracellular matrix environment provides stratum and accompanying signals for cancer cell motility and invasive phenotypes.

One of the first events of mammary gland involution is the initiation of epithelial cell apoptosis. (Figure 2A, upper panel). With weaning, the majority of epithelial cells undergo cathepsin and caspase mediated cell death [50,51], as a means to remove the mature milk secreting cells. Multiple signaling pathways are involved in initiating epithelial cell death during involution, including Janus kinase 1 (JAK1), leukemia inhibitory factor (LIF), dedicator of cytokinesis 1 (Dock1), nuclear factor κ-light chain enhancer of B cells/tumor necrosis factor (NFκB/TNF), and most relevant for the purposes of this review, TGF-β. Impairment in any of these pathways can result in delayed involution [6,52,53,54,55]. Over the past 20 years of investigation, many insightful experiments have been performed to elucidate the timing and significance of TGF-β activation in facilitating mammary gland involution. TGF-β cytokine signaling can be initiated by expression of any of three functionally identical TGF-β genes, which differ in cellular and developmental levels of expression. By mRNA and protein analyses, TGF-β3 is the most abundant subtype of TGF-β in the mouse mammary gland and levels also increase sharply with weaning [6,7,39]. Epithelial sources of TGF-β3 were determined to be sufficient to support epithelial cell apoptosis, through experimental implementation of a conditional transgenic mouse in which *Tgfb3* overexpression in mammary epithelium was driven by the β-lactoglobulin promoter [6]. In this model, at day 1 of involution, overexpression of *Tgfb3* in the epithelial compartment increased epithelial cell apoptosis. Importantly the epithelial cells themselves illustrated nuclear localization of Smad4, emphasizing the potential importance of autocrine canonical TGF-β signaling in epithelial cell death [6]. Mechanistically, recent studies have shown the miR-424/503 cluster, which can be upregulated downstream of canonical TGF-β-Smad activation, participates in mammary epithelial cell death during involution by means of B-cell lymphoma 2 (BCL-2) and insulin-like growth factor 1 (IGF1) receptor downregulation [56,57]. Collectively, these studies provide detailed evidence of the active participation of TGF-β signaling during the initiation of involution.

To more directly assess the unique role of TGF-β during involution, additional evidence is required, for example, by deleting either the gene for the TGF-β cytokine or the TGF-βR. Unfortunately, loss of TGF-β function by gene knockout (KO) is difficult to address, as TGF-β is needed for normal embryonic development and fetal survival, with *Tgfb3* KO mice living for approximately two weeks after birth before succumbing to severe pulmonary abnormalities [58,59]. Furthermore, because of TGF-β’s broad systemic influence, to glean the importance of TGF-β in specific events, more sophisticated experiments must be devised that relegate TGF-β signaling alterations to a specific tissue and/or during a particular window of interest. A novel mammary gland transplantation model was devised to circumvent this limitation, permitting the evaluation of TGF-β function in the post-neonate mammary gland [6]. In this model, mammary glands of newborn pups carrying a null mutation in the *Tgfb3* gene were harvested and placed into wild-type females whose mammary glands were removed before transplantation. Loss of the *Tgfb3* gene within the mammary gland did not impact pubertal gland development or pregnancy, however, loss of *Tgfb3* did result in a three-fold decrease in epithelial cell apoptosis at day 1 post-weaning [6]. Similar results were also obtained in an epithelial lineage specific and temporally controlled conditional KO mouse model in which floxed *Tgfbr2* was ablated by a Whey Acidic Protein (WAP) promoter driven-Cre transgene, resulting in temporal deletion of TGF-βRII within mammary epithelial cells starting at lactation. In this model, mammary epithelial cell specific *Tgfbr2* loss resulted in prolonged lactation and delayed epithelial cell death upon weaning, data consistent with roles for TGF-β signaling in the initiation of involution as well as epithelial cell death [60]. In summary, TGF-β has been discovered to be a necessary player in mammary gland involution and justifies involution as a strong model for understanding many TGF-β signaling processes.

TGF-β is not only involved in the induction of epithelial cell apoptosis during involution, but also facilitates the clearance of the dying epithelial cells (Figure 2A, upper panel). During weaning, apoptotic epithelial cells are removed primarily within the first 72 h of involution, and somewhat surprisingly, clearance is largely accomplished by epithelial cells that do not succumb to early cell death, but rather become phagocytic [61]. Recently, TGF-β3 has been shown to be sufficient to induce a phagocytic phenotype in mature mammary epithelial cells cultured under conditions that mimic epithelial tight junction closure unique to the lactating gland [7]. In this model, TGF-β3 treatment led to γ-secretase induced cleavage of E-cadherin followed by β-catenin nuclear localization. This process resulted in downregulation of cell junction genes including cadherins and induction of a phagocytic phenotype in the epithelial cells [7]. TGF-β3 induced mammary epithelial cell phagocytosis was characterized by loss of adherens junctions but was independent of EMT, although EMT is a common transformed epithelium response to TGF-β (Figure 2A). Rather, in these normal, phagocytic mammary epithelial cells, the EMT associated genes, such as *Snail1*, *Snail2*, *Twist1*, *Twist2*, *Zeb1*, *Vimentin,* and *Fibronectin1* were not increased with TGF-β3 treatment [7]. The function of TGF-β in inducing epithelial cell phagocytosis is not restricted to the mammary gland and was first reported in human retinal pigment epithelial cells [62], illustrating a rarely appreciated but potentially more broadly conserved role of TGF-β in epithelial cells. The consequences of TGF-β induced epithelial cell apoptosis and phagocytosis during mammary gland involution provides one potential mechanism by which the involution window may be tumor suppressive, i.e., by eliminating potential targets of transformation (Figure 2A). However, cell death and phagocytosis have effects on the mammary tissue microenvironment not solely intrinsic to alterations in the epithelial cell compartment that must also be considered, including immune modulation. 

The process of apoptosis actively suppresses the pro-inflammatory arm of the immune system [63]. Specifically, uptake of apoptotic cells by professional cell phagocytes, including macrophages, induces TGF-β production that directly mediates immune suppression [64,65,66]. During involution, immune suppression appears to be an important part of the developmental process that allows for careful restructuring of the normal mammary gland to its baseline, non-secretory state [67]. If cancer cells are present during involution and do not undergo apoptosis, these tumor cells could gain an advantage due to impaired anti-tumor immunity. Thus, this intimate link between TGF-β induced apoptosis in epithelial cells and immunosuppression provides one example of how TGF-β can elicit, at the same time, tumor suppressive and promotional activities, a concept explored more fully below.

## 5. TGF-β in Developmental Immune Tolerance and the Inhibition of Cytolytic Immunity

Throughout all tissues, TGF-β has several discrete immune modulatory functions that overall serve to suppress destructive immune activation that could compromise host viability and/or disrupt finely orchestrated developmental processes. Within the lymphoid branch of immunity, TGF-β is known to directly impair mature T cell proliferation. In immature and naïve T cells, TGF-β also initiates upregulation of the transcription factor FOXP3, which drives differentiation of “regulatory” T cells (Tregs) [68]. Tregs actively impair the actions of cytotoxic (Th1) and cytolytic (Tc1) T cells and are therefore critical for maintaining immune tolerance and tissue homeostasis. Loss of Tregs alone is sufficient to result in devastating multi-organ autoimmunity [69]. In the myeloid branch of immunity, TGF-β stimulates chemotaxis of immature monocytes [70] and plays an important role in the polarization and differentiation of immature myeloid cells to what is referred to as an “M2” macrophage phenotype [71,72,73]. As a result of the M2 program, macrophages produce the cytokines interleukin-4 (IL-4) and interleukin-13 (IL-13) [74]. These cytokines then further polarize the tissue microenvironment by driving differentiation of naïve T cells to a Th2 differentiated state. Molecularly, the Th2 state is achieved through activation of the transcription factor GATA3 [75] chiefly consequent of the signal transducer and activator of transcription 6 (STAT6) activation initiated by signaling via IL-4 and IL-13 receptors [76]. These TGF-β-induced M2, Treg, and Th2 cell populations all secrete TGF-β and interleukin-10 (IL-10), further enhancing suppression of damaging pro-inflammatory and immune cytolytic functions, and instead facilitate tissue rebuilding/remodeling processes. 

**Figure 3:**
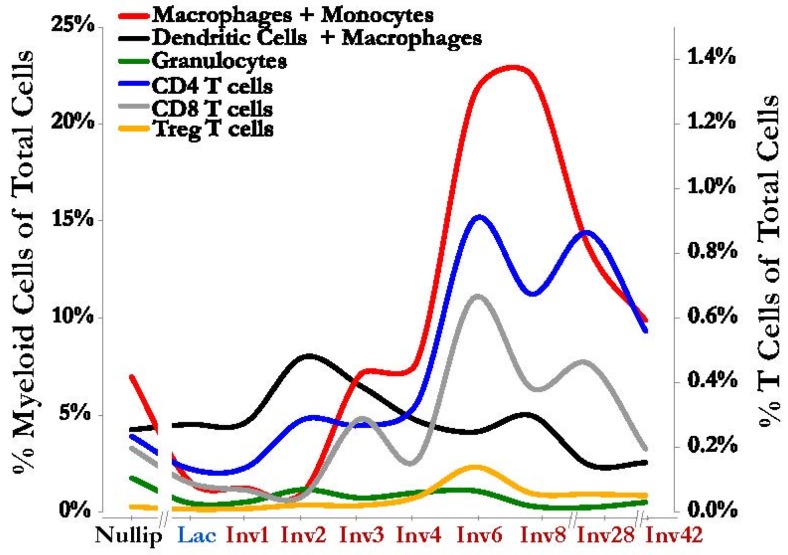
Immune cells increase in the mammary gland during involution. An influx of immune cells consistent with classic wound healing is observed during mammary gland involution including various myeloid cell populations (left axis): Macrophages and monocytes (CD45^+^ Gr1^intermediate/low^ F480^+^ CD11b+, red line); dendritic cells and macrophages (CD45+ CD11c+ MHCII+, black line); and granulocytes (CD45^+^ Gr1^high^ F480^−^ CD11b^+^, green line). T cells are also increased in the mammary gland during involution (right axis): CD4 T cells (CD45^+^ CD3^+^ CD4^+^, blue line), CD8 T cells (CD45^+^ CD3^+^ CD8^+^, gray line) and the immunosuppressive Treg T cells (CD45^+^ CD3^+^ CD4^+^ FoxP3^+^ CD25^+^, orange line). On the *X*-axis, the involution window is labeled in red, as “Inv” followed by a number for the day post-weaning. Both axes represent frequencies of indicated cell populations as a fraction of total cells from the gland, as determined by single cell suspensions analyzed by flow cytometry. The Figure is derived from data reported in Reference [45].

In the post-weaning mammary gland, we and others have reported dramatic temporal changes in the abundance and types of the TGF-β dependent immune cells discussed above [37,39,45,77] (Figure 3). During involution we have observed an increase in number and proportion of regulatory T cells, immature myeloid cells, and M2 macrophages. Consistent with these observations we also noted increases in the immunosuppressive cytokines IL-4, IL-13, and IL-10 during involution [43,45] (Figure 2B). Rather surprisingly, macrophage/monocyte derived cells are not only present at an increased frequency but were also found necessary for the initiation of involution itself [67], yet again illustrating a finely orchestrated relationship between immunity and this developmentally regulated, tissue remodeling process. Cumulatively, these immune profiling data are consistent with expected roles in developmental tissue reconstruction and promoting immune tolerance.

The link between the coordinated death that occurs in the mammary gland with weaning and immune suppression likely exists to minimize self-antigen exposure to tissue resident and local lymphatic immune cells. It could be argued that establishment or maintenance of immune tolerance to these self-antigens would be of paramount importance for subsequent nursing as well as long-term host survival. While not yet formally demonstrated in the mammary gland, inference drawn from other locations throughout the body is consistent with a role for regulatory T cell subsets mediating this protection via TGF-β signaling. Cumulatively, the observed increases in IL-10, immature myeloid cells, regulatory T cells, and M2 macrophages would predict that the immune environment during involution is staged to hinder immunity. While these TGF-β associated immune processes would seem ideal in the involuting mammary gland, they may come at the cost of actively impairing anti-tumor immunity [77,78,79] (Figure 2B).

## 6. The Immune Environment of Involution Is Tumor Promotional and Targetable

Utilizing the mouse model of mammary gland involution, we have shown the involuting mammary microenvironment supports tumor progression significantly more than the mammary gland of the nulliparous host [48,80,81]. This feature is conserved in fully immune competent mice [45], demonstrating that an intact immune system does not mount a sufficient anti-tumor immune response during the involution window. Furthermore, we sought to determine whether the immune phenotypes characterized during involution where carried forward into the tumor microenvironment. We observed tumors injected into immune competent animals during the TGF-β rich involution window are surrounded and infiltrated with increased numbers of immature myeloid cells, M2 macrophages, and T cells expressing IL-10 compared to their nulliparous counterparts; an immune signature associated with pro-tumor immunity. Moreover, IL-10 blockade through antibody administration reduced the observed growth advantage compared to genetically identical tumors implanted into nulliparous hosts, demonstrating the presence of a tumor promotional immune response in the involuting gland [45]. This effective pathway-targeted approach to thwarting specific TGF-β driven immune effects was derived by considering the biology of the entire mammary gland microenvironment. This approach is important, as we have mentioned previously that global TGF-β inhibition has proven to be challenging, likely due to confounding, tissue-level TGF-β signals. With increasing knowledge from within immunity alone, these TGF-β centric interventional outcomes might be predictable, as TGF-β’s impact on immune cell function is also both pro- and anti-tumor, depending upon tissue context.

## 7. TGF-β and Th17 Promote Barrier Function, Reducing Inflammation and Tumor Initiation 

Considering only the TGF-β meditated M2/Th2 biology described above, blockade of TGF-β signaling would be predicted to enhance anti-tumor immunity. However, in mice where TGF-β signaling is impaired in the gastrointestinal (GI) tract, gastric tumors arise consequent of a reinvigorated, robust and chronic Th1 pro-inflammatory immune response to luminal bacteria. This inflammatory milieu leads to elevated levels of carcinogenic reactive oxygen species that repeatedly induce epithelial cell genomic damage promoting tumor initiation and tumor evolution [82,83,84]. The direct mechanisms by which TGF-β mediates this tumor suppressive role have only recently been brought into the light and in so doing further illustrate the power of needing to understand TGF-β signaling in the tissue context, with the study of mucosal borders such as the intestine, leading the field.

At the mucosal borders in the intestine and lung, TGF-β is generated by innate immune cell (i.e., dendritic cells, monocytes) sampling of the luminal bacterial microenvironment [85,86,87]. The resulting TGF-β signals to T cells, instigating upregulation of the transcription factor RORγt [88,89], to generate yet another distinct CD4+ helper T cell subset, Th17s. RORγt in Th17s induces the production of cytokines interleukin-17 (IL-17) and interleukin-22 (IL-22), and as an exquisite example of immune cell-epithelial cell crosstalk, IL-17 and IL-22 stimulate receptors on mucosal epithelial cells promoting epithelial border functionality. Specifically, this TGF-β-mediated cellular crosstalk results in epithelial stem cell maintenance, enhancement of epithelial cell junction integrity, and epithelial cell secretion of mucins and defensin production [85,87]. All of these mechanisms within the mucosal organ barricade against bacterial activation of Th1 immune cells preventing the instigation of the tissue-destructive and tumor initiating pro-inflammatory cycle.

While not classically considered a mucosal organ, the mammary gland displays several mucosal characteristics, especially during lactation and involution. Like the lung and gastrointestinal tract that excrete lipid rich mucus, the lactating mammary gland becomes an excretory organ expressing lipid rich milk. Furthermore, immunoglobulin A (IgA) antibodies and the IgA antibody secreting B cells, known to constitutively populate the GI tract and lung, are uniquely enriched in the mammary gland during lactation and early involution [90,91]. A hallmark feature of a mucosal organ is the role that organ plays as an interface between the outside environment and the inner tissue of the body. During lactation, through the nipple aperture and infant suckling, bacteria routinely interface with luminal epithelial cells, which must serve a barrier function to prevent systemic infection. Lastly, and most relevant to this discussion of TGF-β biology, the TGF-β driven Th17 immune milieu dominates the GI and pulmonary homeostatic state. Consistent with this mucosal characteristic, within hours of bacterial introduction into the duct of lactating ruminants, robust increases of IL-17 and IL-22 are observed [92]. This rapid exacerbation of Th17 cytokines strongly suggests a steady state presence of differentiated Th17 cells in the mammary gland during this developmental window. However, identification of Th17 cells themselves has yet to be reported in the lactating or involuting mammary gland in rodents or humans. Apart from this homeostatic and anti-Th1 induced cancer initiation role at mucosal surfaces, M2, Th2, and Th17 cells work alongside other cell types such as fibroblasts to integrate tissue-level TGF-β signaling [93,94]. The TGF-β mediated roles in fibroblasts also carry with them duplicitous implications for cancer biology.

## 8. The Role of TGF-β in Tissue Repair and Remodeling

As discussed above, TGF-β mediates key events in the early stages of involution including cell death and early immune infiltration and immunosuppression. A later step of involution includes the concerted action of TGF-β to bring about tissue repair and remodeling, with fibroblasts being logically implicated given their well described function in cutaneous wound healing. Fibroblasts are cells of mesenchymal origin found in all tissues, where they support epithelium, in part, by secreting the ECM compartment of the tissue. Under homeostatic conditions fibroblasts proliferate very slowly, if at all, and are involved in basal levels of ECM turnover [95]. If the tissue is damaged, fibroblasts become activated, including the upregulation of motility-, contractility-, ECM synthesis-, and growth factor secretion-programs, all of which facilitate tissue repair. A major regulator of fibroblast activation is the TGF-β family of growth factors [96,97]. TGF-β is normally sequestered in the ECM in an inactive state. Upon cutaneous tissue injury, various proteases including plasmin and matrix metalloproteinase 9 (MMP9) lead to cleavage of Latency Associated Peptide (LAP) resulting in the release of active TGF-β into the local microenvironment [98,99,100]. Active TGF-β can lead to the transcriptional up-regulation of genes involved in fibroblast activation. In the specific example of cutaneous wound healing, it has been shown that TGF-β is necessary for development of the activated fibroblast, termed the myofibroblast. TGF-β-induced myofibroblasts express alpha smooth muscle actin (α-SMA), are motile, deposit collagen-rich ECM, and generate contractile force to facilitate wound closure [101,102,103]. Myofibroblasts under the guide of TGF-β, also deposit tenascin-C and the fibronectin (FN) splice variant ED-A FN, which are both specialized ECM proteins associated with adult tissue repair and embryonic development [102,104]. We next discuss how the latter stages of mammary gland involution share striking similarity with TGF-β-mediated cutaneous wound healing in the types of ECMs and ECM remodeling that occurs.

While it is not known if TGF-β directs fibroblast-mediated tissue repair during mammary gland involution, similarities between TGF-β-mediated cutaneous wound healing and mammary gland involution are evident. For example fibrillar collagen, ED-A FN, FN fragments, matrix metalloproteinases (MMPs), and tenascin-C all increase during involution [43,44,48,105,106,107]. Altogether the similar composition and function of ECM proteins and proteases between TGF-β orchestrated classical cutaneous wound healing and mammary gland involution support the notion that a hard-wired developmental process supports the return to homeostatic conditions in untransformed epithelium. However, these same wound healing programs and ECM depositions are known to trigger aggressive tumor behaviors.

## 9. Dual Effects of ECM and Fibroblasts on Cancer Progression

It has long been appreciated that stromal composition is similar between cancer and healing wounds [108]. We would add that normal mammary gland involution also shares similarities to these unique tissue microenvironments [43,44,48,105,106,107]. As discussed above, wound healing ECM supports untransformed epithelial cell survival and movement during healing, and this paradigm can be exploited by a tumor cell. For example, the de novo deposition of collagen I can literally serve as “tracks” by which cancer cells traffic, leading to local tumor cell invasion and eventual metastasis [109,110]. Importantly, this means that the same biological mechanism can lead to disparate outcomes based on the transformation state of the epithelium, and supports the idea that the activated fibroblast phenotype can be parlayed into a tumor-promotional function when transformed epithelium is present (Figure 2C). However, fibroblasts are no exception to the duality of TGF-β effects, as TGF-β has also been shown to be necessary for fibroblast mediated tumor suppression.

The finding that loss of TGF-β stimulation in fibroblasts supports tumors appears contrary to the known tumor promotional role of activated fibroblasts. Specifically, it has been shown that deletion of *Tgfbr2* in fibroblasts, and thus downstream TGF-β signaling, induces paracrine hepatocyte growth factor (HGF) signaling and promotion of mammary carcinogenesis [111]. Similarly, other studies in a mouse mammary tumor virus driven Polyoma Virus middle T antigen (MMTV–PyVmT) tumor model have shown that loss of one *Tgfbr2* allele in fibroblasts can induce early tumor onset and increased metastasis [112]. Further, it has been shown in vitro, in a fibroblast specific protein (FSP) driven *Tgfbr2* KO *(Tgfbr2*^FSPKO^) model, the fibroblasts upregulate SDF1 [112], which is a molecule that promotes tumor progression directly by inducing tumor cell proliferation and migration, and indirectly via induction of immunosuppressive myeloid-derived suppressor cells [113,114,115,116]. *Tgfbr2*^FSPKO^ tissue also has increased production of various inflammatory mediators including inducible nitrogen oxide synthase, cyclooxygenase-2 (COX2), and NFκB in both epithelial and stromal cells [117]. In accordance with these observations, mice with *Tgfbr2*^FSPKO^ fibroblasts had an increased infiltration of immunosuppressive myeloid-derived suppressor cells and Th17 cells [117], implicating fibroblasts in modulating the immune milieu via TGF-β. 

A striking observation of the studies described above is that *Tgfbr2* KO fibroblasts exhibit a loss of a TGF-β negative feed-back loop, overproducing TGF-β in response to a loss of TGF-βR signaling. This results in increased TGF-β within the tissue that can influence neighboring tumor cells and immune cells directly, elevating tumor aggressiveness [118]. Of note, if these experiments had been performed in simple cell culture systems, or in the absence of either relevant epithelial or immune cell populations, the powerful observation that fibroblasts lacking TGF-βR make more TGF-β would never have been made. In the tissue context of the conditional KO system, the data suggest fibroblasts have a unique and central role in both sensing and modulating the levels of TGF-β in the whole tissue system. Therefore, fibroblast TGF-β signaling may occupy a central role in the instigation of the TGF-β paradox.

## 10. Implications/Next Steps for Therapy 

It has been proposed that in cancers where the TGF-β signaling pathway is mutated, yet TGF-β inhibitors are effective, that this may be due to tumor extrinsic targeting of the fibroblasts and/or the immune milieu [31]. Yet, in the context of the immune system, TGF-β inhibitors would also be predicted to enhanced tumor initiation consequent of elevating the pro-inflammatory and pro-carcinogenic Th1 programs. Hence, from an immunologic perspective, TGF-β inhibitors are likely not appropriate for use as a preventative measure. Somewhere along the continuum of tumor development, blocking TGF-β is expected to reduce immunosuppression providing a reduction in existing tumor burden, however when during cancer progression anti-TGF-β therapy would be detrimental or helpful remains unknown, making this approach risky. Similarly, within the isolated context of the tumor-associated fibroblast, TGF-β inhibition is expected to decrease tumor supportive fibrotic stroma. However, we know that fibroblasts lacking the ability to respond to TGF-β (*Tgfbr2* KO) produce more pro-tumorigenic cytokines. Thus, even though an anti-TGF-β effect may result in the fibroblast itself becoming less pro-tumorigenic, the global effect may be enhanced tumor cell progression, the undesired effect. These evidences outline the fragile nature of direct TGF-β inhibition as related to the microenvironment. 

**Figure 4:**
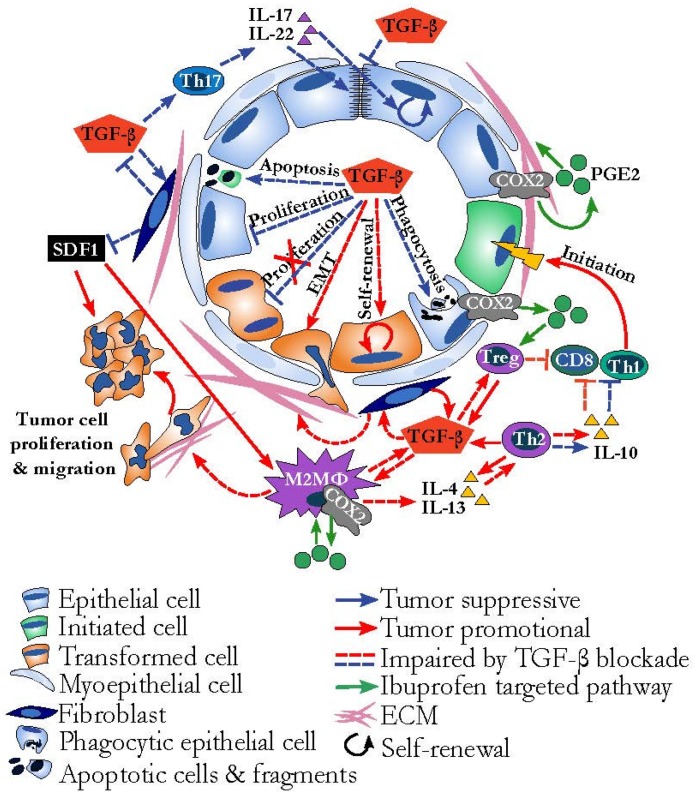
Tissue-level TGF-β signaling and implications for TGF-β targeted therapy. TGF-β can simultaneously mediate pro-tumor (red arrows) and anti-tumor (blue arrows) activities, depending on responding cell type. The effect of an anti-TGF-β therapy would be expected to result in both dampened pro-tumor (red dotted lines) and dampened anti-tumor (blue dotted lines) activities of TGF-β, resulting in mixed tumor outcome. Anti-tumor effects of blocking TGF-β are decreased tumor cell proliferation, M2 polarization, and extracellular matrix (ECM) deposition, and pro-tumorigenic effects include pro-inflammatory tumor initiation (green cell) and enhanced fibroblast production of pro-tumor stromal cell-derived factor 1 (SDF1) and TGF-β. An alternative strategy to direct TGF-β targeting may be to target downstream effectors. TGF-β is known to induce cyclooxygenase-2 (COX2) and subsequent prostaglandin E2 (PGE2) (green circle) production by a number of cell types. Targeting COX2 activity (processes depicted by green arrows) targets pathways in the established cancer environment that may have more uni-directionally pro-tumor effects, such as M2/Th2 polarization and ECM deposition, without impairing TGF-β mediated tumor suppressive functions.

Although there may be success with direct TGF-β inhibition, we propose the employment of alternative strategies. One potential approach is via targeting processes that TGF-β drives that are pro-tumor without directly inhibiting TGF-β, such as the use of cyclooxygenase (COX) inhibitors. TGF-β has been shown to directly upregulate COX2 expression on multiple cell subtypes and many of the pathological features of TGF-β observed in the tumor microenvironment are at least partially attributable to the presence of the COX2 product prostaglandin E2 (PGE2) [63,119,120,121,122,123]. COX inhibitors have been shown to dampen the immunosuppressive pro-tumor behavior of various immune cells [124,125], and also decrease fibrotic pro-tumor stroma [44,48,80,81,126] and could be explored further as an approach to inhibit pro-tumorigenic aspects of TGF-β signaling while preserving anti-tumor attributes (Figure 4). 

In considering where to utilize these hypothetical therapeutic approaches generated by insight from the tissue microenvironmental model, it seems logical to start with tumor environments that employ TGF-β mediated processes. We therefore return our attention to breast cancer and a unique subpopulation of patients who are diagnosed with breast cancer as young women in their reproductive years. Pregnancy associated breast cancer (PABC) encapsulates a younger demographic of breast cancer patients whose breast cancer is diagnosed during pregnancy or within 5 years of having given birth to a child [49]. Amongst this group, it has been determined that patients diagnosed with breast cancer within 5 years of last having a child, but not during pregnancy, have much poorer prognosis, equivalent or worse than the most deadly subtype of breast cancer, triple negative breast cancer [127,128]. This poor prognostic breast cancer is referred to as postpartum breast cancer. Through insights gleaned from multiple rodent models of postpartum breast cancer, it has been proposed that the poorer outcomes experienced by postpartum patients is consequent of indolent tumors being promoted by breast involution, which, as described above, is under the control of TGF-β. Further, in these rodent models of postpartum breast cancer, COX-2 inhibition reduces involution instigated tumor promotion through a reduction in many of the TGF-β mediated processes we have described [48,80] and offers an example of a novel, targeted and efficacious therapeutic approach that might be adopted for a patient cohort for whom standard of care is poorly effective. 

Many biological processes mediated by TGF-β have been implicated in both cancer suppression and promotion, highlighting TGF-β’s central position in the cancer field. With the current depth of mechanistic understanding, we advocate for shifting the focus of TGF-β mediated research to include not only other cellular actors from the microenvironment but also utilizing relevant microenvironments themselves where all the actors of TGF-β are present. This would allow researchers to quantify how the diverse functionalities of TGF-β within different cell subsets compete or reinforce one another to impact cancer outcomes. Furthermore, we encourage investigation downstream of TGF-β signaling to elucidate specific processes dependent upon TGF-β, as downstream targets might bias treatment more powerfully towards anti-cancer responses without targeting TGF-β directly. In summary, by utilizing the developmental window of mammary gland involution, we have been able to investigate many of the various roles of TGF-β and have concluded that the paradox of TGF-β is the unresolvable reality of a complex interactive microenvironment. Moving forward we must integrate this complex reality into therapeutic strategies to more predictably improve patient outcomes.
